# Clinical Characteristics, Investigations and Treatment in Children with Chronic Urticaria: An Observational Study

**DOI:** 10.3390/medicina60050704

**Published:** 2024-04-25

**Authors:** Enrico Vito Buono, Giuliana Giannì, Sara Scavone, Carlo Caffarelli

**Affiliations:** Clinica Pediatrica, Azienda Ospedaliero-Universitaria, Department of Medicine and Surgery, University of Parma, Via Gramsci 14, 43126 Parma, Italy; enricovito@libero.it (E.V.B.); giannigiuliana90@gmail.com (G.G.); sara.scavone92@gmail.com (S.S.)

**Keywords:** chronic urticaria, children, allergic disease, urticaria treatment, omalizumab, urticaria diagnosis

## Abstract

*Background and Objectives:* The guidelines for chronic urticaria in children contain recommendations that are often based on adult studies. The diagnostic pathway has not been standardized and the effectiveness of anti-H1, omalizumab, montelukast, and systemic glucocorticoids is rarely reported in the pediatric population. There is a wide variation in the rate of remission of chronic urticaria between studies. The aim of this study is to enhance our understanding of pediatric chronic urticaria. *Materials and Methods:* This study enrolled 37 children with chronic urticaria aged from 0 to 18 years. Demographic parameters, medical history, clinical features, laboratory data and treatment information were collected. Children were treated with the recommended dosage of second-generation H1-antihistamines, which was increased by up to twofold. Omalizumab was added for refractory anti-H1 patients. A three-day course with systemic glucocorticoids was administered for severe exacerbations. Montelukast was administered to some children. *Results*: Wheals without angioedema were common. Chronic urticaria was spontaneous in 32 children (86.48%), inducible in 2 (5.41%), induced by a parasite in 1 and vasculitic in 2. Treatment of the potential causes of chronic urticaria was of no benefit, except for eradication of Dientamoeba fragilis. Chronic urticaria was resolved within three years in 45.9% of cases. Allergic diseases were present in nine children (24.32%) and autoimmune diseases were present in three (8.11%). All children were treated with anti-H1 at the licensed dose or at a higher dose. A partial or complete response to anti-H1 was observed in 29 (78.38%) patients. Montelukast showed no benefit. All children treated with omalizumab responded. Systemic glucocorticoids were successfully used to treat exacerbations. *Conclusions:* Our findings indicate that laboratory tests should not be routinely performed in children with chronic urticaria without clinical suspicion. However, comorbidities such as thyroid autoimmune disease and coeliac disease are suggested to be monitored over the chronic urticaria course. These clinical conditions could be diagnosed from the diagnostic framework of chronic urticaria. Increasing the dosage of anti-H1 and omalizumab was effective in children resistant to standard treatment but we still need further studies to generate a standard patient-centered treatment.

## 1. Introduction

Chronic urticaria (CU) presents with wheals, angioedema (AE) or both, lasting over six weeks. Wheals appear as localized swelling with an erythematous basis and differences in size and shape; they are itchy, fleeting and usually last for 24 h. In urticaria related to vasculitis and pressure, wheals persist for longer. AE affects the deeper layers of the dermis, presenting itself as a submucosal or subcutaneous swelling of areas, which usually involves the face, especially the lips and eyelids, the back of the hands and feet and the genital area. AE usually causes pain and a burning sensation and it is not itchy. It lasts 2–3 days [1]. The etiopathogenesis is often unclear and this is related to inappropriate diagnostic investigations and specialist consultations. CU is classified as chronic spontaneous urticaria (CSU) when there is no specific precipitating trigger detectable, and chronic inducible urticaria (CIU) if it is elicited by one or more triggers [2,3]. In childhood, CSU is the most common type of CU and has been reported in 60–90% of cases. IgE-mediated Type I and Type IIb autoimmunity is often involved in CSU, with unclear incidence [1,2]. Some studies distinguish between patients with and without positive autologous serum skin tests (ASSTs) or other laboratory indicators of an autoimmune process. If it is not accompanied by other systemic symptoms detectable by clinicians, CSU is rarely a sign of an underlying disease [2,3]. The rate of autoimmune thyroiditis and coeliac disease [4,5] or allergic diseases is undetermined in children with CU. The frequency of CIU in children with CU greatly varies from 22% to 40% among studies [6,7,8]. Dermographism, cholinergic urticaria and cold urticaria are the most common triggers of CIU [7,9,10]. Food allergy is a rare cause of CIU in childhood, with a prevalence of 0–8.6% [6,7,8,11,12]. There are some data in studies involving adults to be confirmed in children that show the role of history and physical examinations in differentiation of CSU from CIU [13] and the process for selection of the diagnostic tests that are needed to confirm CIU [1,3,14] and associated diseases. The prognosis of CU is generally good, but it is often long-lasting. In pediatric cohorts, there is a wide variation in the rate of remission of CU between studies. It fluctuates from 10% to 32% of cases within one year, from 31% to 54% within three years and from 38% to 72% within five years [6,7,15]. Moreover, in the time frame of exacerbations of symptoms, the impairment of the quality of life of affected children is not neglectable. A licensed dose of second-generation H1-antihistamines is usually the first-choice treatment, with the possibility of increasing the dose by four times the basal dosage [2,16]. In patients resistant to anti-H1, many national regulatory agencies have approved the use of omalizumab, a monoclonal anti-IgE antibody, in adolescents >12 years of age [17,18]. In patients with an age <12 years, data concerning the efficacy and the safety of omalizumab are lacking. Real-life data published on the efficacy of increasing the anti-H1 dosage, using omalizumab and other therapeutic options such as a short course of systemic glucocorticoids during severe CU exacerbations [2,19] and montelukast [20,21], are rarely reported. Overall, the recommendations of guidelines for CU in children [2] are often based on adult studies since there is a lack of clinical data in children. So, their appropriateness remains uncertain. The aim of this study is to analyze the clinical characteristics, duration, underlying causes, comorbidities and treatment options for children with CU to address this challenge and enhance our understanding of CU in children.

## 2. Materials and Methods

The study population included children, aged from 0 to 18 years, with chronic urticaria lasting at least 6 weeks from the Pediatric Allergy Unit of the University Hospital of Parma in Italy. Patients with a diagnosis that was inconsistent with the history and physical examination, those with acute or recurrent acute urticaria (symptoms lasting less than 6 weeks), those with missing information on the report format or those whose follow-up results could not be obtained were excluded from the study. Patients were visited at the baseline and then they were visited every 1 or 3 months until resolution of symptoms. Data were retrospectively collected. They included demographic parameters, medical history, clinical features (age of onset of CU, triggers, course of symptoms, site of wheals/angioedema, duration of disease, urticaria control test (UCT), and the Urticaria Activity Score 7 (UAS7) to assess severity of itching and wheals in the last four weeks), and other associated symptoms. In agreement with the guidelines [2], in all children, a blood sample was taken for the following tests: white cell count, C-Reactive Protein (CRP)/erythrocyte sedimentation rate (ESR), serum antibodies to *Mycoplasma pneumoniae* and the Epstein–Barr virus, thyroid-stimulating hormone (TSH) and unbound thyroxine (T4) levels, screening for coeliac disease (anti-Tissue Transglutaminase antibodies, anti-endomysial antibodies, IgA). Skin prick tests (SPTs) for food allergens were performed for all children. A panel with the following extracts was performed: wheat, rice, cod, lamb, beef, chicken, tomato, carrot, potato, peanut, bean, pea, peach and walnut. Selected children underwent an SPT to fresh food and/or aeroallergens to confirm a clinical suspicion. A battery of inhalant allergens (*Dermatophagoides farinae*, *Dermatophagoides pteronyssinus*, grasses, alternaria, aspergillus, animal epithelium (cat, dog), olive, birch, hazelnut, English plantain, pellitory, mugwort, ambrosia) was used. Histamine was used as a positive control, and 0.9% saline was used as a negative control. SPTs were evaluated 15 min after application. A wheal diameter 3 mm greater than the negative control was considered positive. Treatment of children treated with H1-antisthamines for other allergic conditions was stopped at least three days before SPTs, as H1-antihistamines interfere with skin responsiveness. When the clinical history suggested a temporal relationship between exposure to a trigger or the onset of a systemic disease and CSU, serum-specific IgE, C4, C1-inhibitor (C1INH), and kidney and liver function tests; a skin biopsy for vasculitis or mastocytosis; a jejunal biopsy for celiac disease; antinuclear antibody (ANA) and IgG anti-thyroid peroxidase tests; a throat swab for Group A Beta-Hemolytic Streptococcus (GABHS); cultures or tests for parasites; tests for circulant antibodies to infectious diseases; and tests for inducible urticaria [2] were performed. Moreover, serum-specific IgE levels to foods or aeroallergens were measured by ImmunoCAP Phadiatop (Thermo Fisher Scientific, Milano, Italy) in patients for whom there was a clinical suspicion of food or inhalant allergies or with clinical manifestations not concordant with SPT results. Specific IgE levels above 0.35 kIU/L were considered positive. An ASST [22] was performed when the aforementioned investigations were negative. An aliquot (50 μL) of autologous serum was injected intradermally into the volar forearm for the suspicion of autoimmune CU. The wheal response was assessed after 30 min. An ASST was positive when a red wheal had a diameter of at least 1.5 mm more than that of the saline control. Vasculitis and cutaneous mastocytosis were diagnosed via a skin biopsy. Children were treated with the recommended dosage of second-generation H1-antihistamines. When treatment was unsuccessful, the anti-H1 dose was increased by up to twofold. Omalizumab (300 mg every 4 weeks) was the add-on treatment in refractory anti-H1 patients. Montelukast was administered to some children before starting omalizumab treatment. A short course of 3 days with systemic glucocorticoids was given for severe urticaria exacerbations. The efficacy of treatments regarding the clinical score was recorded. The study was approved by the local ethics committee.

For the statistics, data are presented as numbers (n) and frequencies (%) if data were categorical, and as means, medians and ranges if they were continuous variables.

## 3. Results

### 3.1. Characteristics of Chronic Urticaria

Thirty-seven children with CU were enrolled. There were 18 (48.6%) male and 19 female patients; the mean age was 8 years and the median was 7.3 years, with a range of 0.5–17.1 years. There were 32 (86%) Caucasian, 3 (8%) Black, and 2 (5%) Asian patients. CU was recurrent in 2 children and continuous in 35. Their history is depicted in Table 1. At baseline, 26 (70.27%) children only had wheals and 27 children had both wheals and angioedema. The mean number of days a week with symptoms was 5.93 (median 7, range 3–7). Wheals were widespread in 11 (29.73%) children, on the trunk and limbs in 7 (18.92%), on the face and limbs in 7 (18.92%), on the face, trunk and upper limbs in 4 (10.81%), on the face and trunk in 2 (5.42%), on the neck, trunk and abdomen in 1 (2.70%), on the face, trunk, hands and feet in 1 (2.70%), on the neck, trunk, upper limbs and thighs in 1 (2.70%), on the face, abdomen, hands and thighs in 1 (2.70%), on the face, trunk and limbs in 1 (2.70%), and on the neck, elbows, forearm and on the back in 1 (2.70%). In one child (2.70%), angioedema involved the lips and ears, in two (5.41%) in involved the face, in two (5.41%) in involved the lips, in one (2.70%) in involved the lip and jaw, in two (5.41%) in involved the eyelids and lips, in one (2.70%) in involved the face and hands, in one (2.70%) in involved the eyelids, lips, hands and feet, and in one (2.70%) in involved the limbs and cheek. Twenty-eight children (75.68%) also experienced itching. It was mild (itching did not disturb) in 6 children (21.42%), moderate (itching was disturbing but it did not interfere with daily activities) in 14 (50%), and intense (itching interfered with daily activities and sleeping) in 4 (14.29%). The intensity was undetermined in four children (14.29%). In 22 children, the mean UAS7 was 22.77 (median 19, range 8–50) and the mean UCT was 4.86 (median 5, range 2–10); the severity of CU at onset is shown in Table 1. No child had absent or controlled (UAS7 0–6) CU. The severity of CU was mild (UAS7 7–15) in seven cases, moderate (UAS7 16–27) in seven cases, and severe (UAS7 28–42) in eight cases. The age at onset of symptoms and at recovery is reported in Table 2.

### 3.2. Underlying Causes

CSU was diagnosed in 32 of 37 children (16 females and 16 males) (86.48%). Two of twenty-three (8.7%) children with CSU (one female and one male) had a positive ASST. Vasculitic CU was diagnosed by biopsy specimens in two females (5.41%). Two children (5.41%) (one male and one female) had CIU. One child had cholinergic CIU elicited by heat and exercise and one child showed isolated dermographism. In one child (2.70%), CU was associated with a Dientamoeba fragilis infection. In our population, celiac disease, Graves’ disease, and viral, bacterial, and parasitic infections coexisted with CU (Table 3). The treatment of such potential causes of CU was of no benefit, except for eradication of Dientamoeba fragilis. No remission of CU was noted following successful treatment of mycoplasma pneumoniae (nine children), Graves’ diseases (one child), celiac disease (one child), and GABHS (one child) or after resolution of acute EBV infections (three children) (Table 3).

Blood counts and differential leucocyte counts were altered in 8/37 (21.6%) children; a high CRP/ESR was observed in 4/37 (10.8%) children. Liver function tests and kidney function tests were performed and serum levels of C4 and C1INH were measured, respectively, in 18, 10, 12, and 9 children, with normal values. A positive ANA test was returned for one child. SPT results were unreliable in three (8.11%) children due to non-specific cutaneous hyperreactivity. SPTs were negative in 10 (27.03%) cases. SPTs were positive for grasses in 14 children (37.84%), for pellitory in 5 (13.51%), for English plantain in 4 (10.81%), for aspergillus in 2 (5.41%), for whole cow’s milk in 8 (21.62%), for alfa-lactalbumin in 5 (13.51%), for beta-lactoglobulin in 4 (10.81%), for casein in 1 (2.70%), for birch in 3 (8.11%), for olive tree in 3 (8.11%), for alternaria in 1 (2.70%), for dermatophagoides farinae in 13 (35.14%), for rice in 3 (8.11%), for dermatophagoides pteronyssinus in 12 (32.43%), for mugwort in 4 (10.81%), for cat epithelium in 5 (13.51%), for hazelnut tree in 4 (10.81%), for dog epithelium in 6 (16.22%), for ambrosia in 3 (8.11%), for egg white in 4 (10.81%), for bean in 2 (5.40%), for hazelnut in 3 (8.11%), for carrot in 2 (5.40%), for wheat in 3 (8.11%), for beef in 1 (2.70%), for tomato in 2 (5.40%), for potato in 1 (2.70%), for peanut in 1 (2.70%), for pea in 1 (2.70%), for peach in 2 (2.70%), for trout in 1 (2.70%), for shrimp in 1 (2.70%), for banana in 1 (2.70%), for soy in 2 (5.40%), for cladosporium in 1 (2.70%), for cod in 1 (2.70%), for egg yolk in 2 (5.40%), and for corn in 1 (2.70%).

Prick tests were performed in three children (8.11%) with positive results for cow’s milk and pumpkin pulp, peel, and seed in one child; positive results for amoxicillin-clavulanate in one child; and positive results for lentils in one child. Specific IgE levels were detected in 32 children (86.49%), while they were negative in 18 children (48.65%). Specific IgE was positive to olive trees in three children (8.11%), to egg white in four (10.81%), to milk in four (10.81%), to DP in three (8.11%), to DF in two (5.41%), to cat epithelium in two (5.41%), to dog epithelium in two (5.40%), to shrimp in one (2.70%), to pellitory in three (8.11%), to mugwort in five (13.51%), to ambrosia in one (2.70%), to birch in two (5.41%), to wheat in two (5.41%), to peanut in one (2.70%), to soybean in one (2.70%), to walnut in one (2.70%), to hazelnut in two (5.41%), to peach in one (2.70%), to alternaria in one (2.70%), and to grass in five (13.51%). One child (2.70%) underwent an oral food challenge to milk with a negative result. We found that exacerbations of CSU were triggered by stress and exposure to house dust and grasses in one child (2.70%); food (hazelnut cream, custard and tomato) in one child (2.70%); and house dust in one child (2.70%). The two children with allergies to house dust mites were treated with house dust mite control measures, and one child received house dust mite immunotherapy, given for a concurrent allergic rhinitis. It was unclear if this treatment was helpful for remittance of CU.

### 3.3. Drug Treatment

Drug treatment is reported in Table 4. No side effects were reported. All children enrolled in the study were treated with a second-generation H1-antihistamine. Cetirizine was prescribed to 29 (78.38%) children. Other second-generation H1-antihistamines prescribed were desloratadine, bilastine, loratadine, and levocetirizine. In most cases (72.97%), we used the standard dose of a second-generation H1-antihistamine. The response to H1-antihistamine therapy was mostly partial (62.16%). The standard dosage improved symptoms in 64% of children, but a complete remission occurred in only 16% of cases. There was a positive response to a two-fold dose in 5 out of 10 children and the lesions completely cleared in two cases. We did not observe that any H1-antihistamine was more effective than others. A short course of systemic glucocorticoids for three days was administered to 13 children (35.14%) during severe CU exacerbations. The most commonly used systemic glucocorticoid was prednisone (69.23%). The response to systemic glucocorticoids was complete in most cases (61.54%). Omalizumab was prescribed in six children with CU resistant to anti-H1. The response after the first course of therapy was good in most cases (83.33%). In about one third of the cases, CU recurred after omalizumab withdrawal, but the response to re-treatment was excellent. We added montelukast in two children (5.41%) who had CU resistant to a second-generation H1-antihistamine. Montelukast was unhelpful. No adverse drug reactions were reported during follow-up.

## 4. Discussion

The aim of the current study was to better characterize CU in children since data on the disease are still scarcely reported. The performed investigations showed that CU was associated with autoimmune disorders, allergic sensitization, infections, and parasite infestation. An important finding of our study is that treatment of such potential causes of CU was not associated with resolution of symptoms, except for eradication of Dientamoeba Fragilis in one child. Furthermore, sensitization to foods or aeroallergens was never a single trigger of CU, but they aggravated symptoms in some cases. We diagnosed CIU at a lower rate in our cohort than that reported in previous studies [6,7,8]. A possible explanation could be related to a different classification of dermographic CU that involves just children with isolated dermographism and not all children with dermographism, because dermographism is a very common sign in children with CU. In our cohort, the rate of CSU was 86%, higher than that reported in previous pediatric studies [2]. So, our findings highlight that for children with CU [13,14], laboratory tests should not be performed to identify the culprit agent without a strong suggestive clinical history. This is in agreement with recommendations in the guidelines [2]. Another focus of this study was the rate of comorbidities. Positive IgE test results to food allergens and/or airborne allergens were commonly reported in patients with CU. Accordingly, we found that 73% of all patients were sensitized to aeroallegens or food allergens. Moreover, the most common comorbidities were allergic diseases, which were found in 24% of cases. The frequency of allergic rhinitis was 21.62%, while asthma was present in 8.11% of patients. In our study, the rate of allergic rhinitis was higher than reported previously, varying from 7% to 17% [23]. This difference may be related to the study population. Indeed, we found that in children with CU, the frequency of allergic rhinitis was similar to that of the local pediatric population without urticaria. Two children were sensitized to house dust mites, and in these patients, CU was resolved with house dust mite control measures and house dust mite immunotherapy. More studies are necessary to clarify whether remission of CU is linked to the treatment of house dust mite allergies or to the natural history of the disease. One child with grass sensitization exhibited exacerbation of urticaria following exposure to seasonal pollens. We found no association of the onset of urticaria with sensitization to foods, apart from one child, whose urticaria was aggravated by the ingestion of some foods or atopic eczema, which is often associated with food allergies [24,25]. Several studies in adults have investigated whether autoimmunity may have a part in the onset of CU. It was thought that the onset of autoimmune diseases in CU might be related to a pathogenetic role of autoimmunity in CU. Data on the pediatric population are scarce. It is of note that in our population, autoimmune diseases, which were diagnosed in three children (diabetes mellitus type 1, Graves’ disease, coeliac disease), were the second most common comorbidity. Our findings are in contrast with studies focusing on adults, showing that association with multiple autoimmune diseases is common. However, our observations are in agreement with other case–control studies showing that the prevalence of autoimmune thyroiditis was 10–30 times higher in children with CSU than in the general population and that of coeliac disease was 8–10 times higher [4,5]. Along this line, the presence of functional autoantibodies in the serum of CSU patients that is revealed by a positive ASST test may indicate an autoimmune pathway of CU. Previous reports identified a positive ASST in 22–53% of patients with CSU [2,11,26]. Our results further minimized the pathogenetic role of autoimmunity in CSU, since we found that ASSTs were positive only in two children with CSU. Regarding the clinical course, we found that the prognosis of pediatric CU was good. The rate of recovery after one and five years from the onset of CU matched with the previous reports [6,7,15], while after three years it was slightly increased compared with previous studies [6,7,15]. The shorter duration of CU could be explained by earlier omalizumab use in children refractory to anti-H1 compared with other reports [23]. All children with CIU were still affected at the end of follow-up, and the duration of the disease was longer than in other children with CSU [9,27]. It is of interest that itching was reported by parents only in 76% of cases. We think that this low frequency may be explained by the fact that in a child, itching is not easily assessed. A further result of the study was about the response to the treatment. Most studies showed that the response rate of children with CU to licensed doses of second-generation H1-antistamines is higher compared to the adult population [26,28,29,30,31]. On the other hand, Ozen B [23] found that only 37% of 141 children responded to standard anti-H1 treatment. In agreement with these findings, we observed that the response of the disease to second-generation H1-antihistamines was partial in 62% of children, while it was complete in just 16% of patients. In agreement with previous studies, our results confirmed recent studies that any H1-antihistamine is more effective than others [2]. Furthermore, we found that an increase in the daily dosage of a second-generation H1-antihistamine may be recommended in children over 12 years of age, refractory to standard dosing [31], since this was safe and effective. However, a beneficial effect of up-dosing was observed in only 50% of CU children, and complete remission was observed in 3 of 10 children, as previously reported (10–25%) [29,30,31]. Our study provides novel data on montelukast, omalizumab, and systemic glucocorticoid treatment in children not responding to anti-H1 antihistamines. In adults, there is weak evidence of montelukast’s efficacy as an add-on treatment to H1-antihistamines [20,21]. In children, data are still lacking. In our study, montelukast was given to two children without any benefit. So far, the single approved biologic for children aged ≥12 years with CSU is omalizumab. Randomized clinical trials on the efficacy of omalizumab in children involved only 39 adolescents with antihistamine-resistant CU [32]. However, there has been a recent increase in real-world studies on the efficacy of omalizumab in childhood [33,34,35]. Dekkers C [33] showed a complete/good response to omalizumab in 76.3% of 38 children aged 3 to 17 years. In a retrospective observational study, Song X-T [34] showed that 66.7% of 12 children aged 3–16 years were complete or well responders at week 4 of the treatment with omalizumab. The results confirmed the findings of the study of Ocak M [35], in which treatment with omalizumab led to a complete response in 89.6% of the pool of 29 children between 12 and 18 years old with CU. Our findings strengthen and extend previous findings. Indeed, we showed that all the patients of the group treated with omalizumab obtained a good or excellent control of symptoms with a good tolerance, corroborating the good profile of efficacy and safety of the use of omalizumab in children affected by histamine-resistant CU. Of note, all children in our cohort maintained the remission of symptoms at the end of omalizumab courses. Furthermore, we were able to show that even if CU recurred after suspension of omalizumab, which occurred in about one third of the cases, the response to re-treatment was excellent [17]. There are no controlled studies on the efficacy of corticosteroids in children with CU, although they have successfully been used in practice [2,19]. We administered systemic glucocorticoids during CU exacerbations in some children and most of them exhibited a complete remission of symptoms.

The most important weakness of our study was the retrospective design and the relatively small population. Another weakness was that an ASST is not enough to diagnose an autoimmune etiology. Urticaria is considered autoimmune when a positive ASST is associated with a positive BHRA and a positive immunoassay for the IgG autoantibodies anti FceRIa and/or anti-IgE (WB or ELISA) [36]. These tests were not performed. So, we are unable to calculate how many children had autoimmune urticaria. Another limitation is that the design of the study did not permit us to reach firm conclusions on the efficacy of the drugs. Finally, some children dropped out during follow-up. CU is a long-term disease and patients generally do not continue follow-up when the condition improves. So, follow-up results were not available for all patients and some data have not been registered. However, the study presents real-life data from a tertiary allergy center and this makes our study valuable.

## 5. Conclusions

Although well described in adults, there are scarce and heterogeneous data on the management of CU in children. So, our findings shed light on the unclear aspects of CU in children and they may help pediatricians in decision making. Our results underpin guideline recommendations [2] that detailed laboratory tests to identify a triggering factor should not be routinely performed in children with CU without clinical suspicion. Indeed, laboratory test results were found to not be linked to CU. However, CU worries parents and patients, who urge physicians to find a cause. Consequently, a significant number of physicians do not follow guidelines [37] and prescribe costly, useless, laboratory tests. Our results show that reassuring parents about the benign nature of the disease is needed more than performing investigations. It is noteworthy that our findings showed that thyroid autoimmune diseases, particularly Hashimoto thyroiditis and coeliac disease, could be diagnosed from CU investigations. Future studies should focus on a long-term prospective screening approach to investigate the influence of associated conditions on CU. Finally, we demonstrated that increasing the dosage of anti-H1 and omalizumab was effective in children resistant to standard anti-H1 treatment. Further controlled studies on larger samples are necessary to select reliable markers of prognosis and to choose the most effective medicines for each patient. The development of new drugs is currently in progress for an easier control of the disease in children.

## Figures and Tables

**Table 1:** Family history, comorbidities and severity.

Characteristics	Frequency
**Family history (n)**	
chronic urticaria	4 (10.81%) [2 mothers with CSU, 1 CSU was autoimmune; 1 mother with CIU (pressure urticaria); 1 father with CU (the type was unknown)]
allergic diseases	29 (78.38%)
autoimmune diseases	4 (10.81%)
**Comorbidity history (n)**	
allergic diseases: rhinitis conjunctivitis asthma hypersensitivity reaction to hexavalent vaccine hypersensitivity reaction to amoxicillin clavulanate	14 (37.84%) 9 (24.32%) 8 (21.62%) 7 (18.92%) 3 (8.11%) 1 (2.70%)
autoimmune diseases: Coeliac disease Graves’ disease Diabetes Type 1	3 (8.11%) 1 (2.70%) 1 (2.70%)
vasculitis cutaneous mastocytosis epilepsy and right schizencephaly esophageal atresia, dystopic tetraparesis, tetralogy of Fallot.	1 (2.70%) 1 (2.70%) 1 (2.70%) 1 (2.70%)
**Severity** Recurrent course/continuous course (n)	2 (5.41%)/35 (94.59%)
Days a week with symptoms (mean, median, range)	5.93 in the 4 weeks before medical examination (7, 3–7); data lacking in 10 (27.03%) clinical cards
Clinical manifestations of patients (n) wheals wheals and angioedema	26 (70.27%) 11 (29.73%)
UAS7 (mean, median, range)	22.77, 19, 8–50
UCT (mean, median, range)	4.86, 5, 2–10
Severity of CU (n) absent CU (UAS7 0) controlled CU (UAS7 1–6) mild CU (UAS7 7–15) moderate CU (UAS7 16–27) severe CU (UAS7 28–42)	0 (0%) 0 (0%) 7 (18.92%) (7 males) 7 (18.92%) (6 females and 1 male) 8 (21.62%) (7 females and 1 male);

**Table 2:** Age at onset and duration of chronic urticaria (CU).

Characteristics	Frequency
Age at onset, years (mean, median, range) (*n* = 37)	8, 7.3, 0.5–17.1
Age at recovery, years (mean, median, range) (*n* = 19)	10.2, 10, 1.9–19
Children dropped out during follow up (*n*)	8 (21.62%)
Children still affected at last visit (*n*)	10 (27.02%)
Duration of CU in still affected children, years (mean, median, range)	4.9, 3.9, 1.7–12
Duration of CU in recovered children, years (mean, median, range)	1.8, 1.3, 0.6–7.3

**Table 3:** Autoimmune diseases, laboratory and physical triggers and their relationship with chronic urticaria.

Variables	Number of Children	CU Remission with Treatment
Graves’ disease	1/37 (2.70%)	0/1
Reduced TSH and high T4 levels	1/35 (2.86%)	
Positive thyroid antibody levels	1/27 (3.70%)	
Coeliac disease	1/37 (2.70%)	0/1
Positive anti-tissue transglutaminase antibody	1/36 (2.78%)	
Positive deamidated gliadin peptide antibodies	1/36 (2.78%)	
Positive throat swab for GABHS	1/6 (16.67%)	0/1
Positive ASO titer	1/1 (100%)	
Positive parasites in stools (n = 18)	1/18 (5.56%) (Dientamoeba Fragilis)	1/1 (100%)
Antibodies to virus		0/3
positive	17/34 (50%)	
IgG anti-VCA (EBV virus)	15/34 (44.11%)	
IgG anti-EA (EBV virus)	7/34 (20.59%)	
IgM anti-VCA (EBV virus)	3/34 (8.82%)	
IgG anti-Parvovirus B19	1/34 (2.94%)	
IgG anti-EBNA	1/34 (2.94%)	
IgG anti-Adenovirus	1/34 (2.94%)	
IgG anti-CMV	1/34 (2.94%)	
Anti-mycoplasma pneumoniae antibodies		0/9
IgG positive	10/32 (31.25%)	
IgM positive	9/32 (28.13%)	
Positive challenge tests for CIU:		
isolated dermographism	1/37 (2.70%)	
ice cube test	0/1	
exercise test	1/1 (2.70%)	

GABHS: group A beta-hemolytic streptococcus, ASO: Antntistreptolysin O, VCA: virus capside antigen, EA: early antigen, EBNA: Epstein–Barr nuclear antigen.

**Table 4:** Drugs for chronic urticaria.

Drug	Number of Children
Second-generation H1-antihistamine	
cetirizine	29/37 (78.38%)
desloratadine	4/37 (10.81%)
bilastine	2/37 (5.41%)
loratadine	1/37 (2.70%)
levocetirizine	1/37 (2.70%)
Dosage of second-generation H1-antihistamine	
standard	27/37 (72.97%)
twofold	10/37 (27.03%)
Response to H1-antistamine therapy	
complete remission (>90%)	6/37 (16.22%) Standard dose: 3 Twofold the dose: 3
partial	23/37 (62.16%) Standard dose: 21 Twofold the dose: 2
unresponsive	8/37 (21.62%) Standard dose: 3 Twofold the dose: 5
Systemic glucocorticoids	13/37 (35.14%)
prednisone	9/13 (69.23%)
betamethasone	4/13 (30.77%)
Response to systemic glucocorticoids	
complete remission	8/13 (61.54%)
partial	5/13 (38.46%)
Omalizumab	6/37 (16.21%)
Response to omalizumab	
Excellent (complete control) after the first course of therapy	1/6 (16.67%)
Good (marked improvement >75% in clinical picture, UAS and UAS7) after the first course of therapy	5/6 (83.33%)
Recurrence after suspension of the first course of therapy:	2/6 (33.33%)
complete control after the first dose of fourth course	1/2 (50%)
2. excellent response with complete remission after second course	1/2 (50%)
Unresponsive	0
Montelukast Response	2/37 (5.41%) 0/2 (0%)

## Data Availability

Data are available from the authors upon request.
